# Esophageal perforation as a complication of dengue fever: a case report

**DOI:** 10.1093/jscr/rjaf948

**Published:** 2025-11-25

**Authors:** Mahmoud Yehya, Adel Abdelhadi, Alyaa Alramah, Rigashy Raghavan, Maaida Sheikh

**Affiliations:** Department of Thoracic Surgery, SAQR Hospital, 155, Al Reydan Street, Al Juwais, Ras Al-Khaimah, PO Box 5450, United Arab Emirates; Department of Intensive Care, SAQR Hospital, 155, Al Reydan Street, Al Juwais, Ras Al-Khaimah, PO Box 5450, United Arab Emirates; Ras Al-Khaimah Medical and Health Sciences University, 155, Al Reydan Street, Al Juwais, Ras Al-Khaimah, PO Box 11172, United Arab Emirates; Ras Al-Khaimah Medical and Health Sciences University, 155, Al Reydan Street, Al Juwais, Ras Al-Khaimah, PO Box 11172, United Arab Emirates; Ras Al-Khaimah Medical and Health Sciences University, 155, Al Reydan Street, Al Juwais, Ras Al-Khaimah, PO Box 11172, United Arab Emirates

**Keywords:** esophageal perforation, Boerhaave syndrome, dengue fever, mediastinitis, case report

## Abstract

Boerhaave syndrome is a rare condition that is often caused by forceful vomiting, often linked to overeating or alcohol intake. Early diagnosis and management are essential to reduce mortality and morbidity. Severe vomiting from any cause may trigger the same mechanism. Dengue fever is a viral illness; besides fever, nausea and vomiting are common. No cases of Boerhaave syndrome as a complication of dengue fever have been reported. We describe a patient with confirmed dengue fever who developed Boerhaave syndrome after severe vomiting and underwent prompt surgical repair with successful recovery. To our knowledge, this presents the first reported case of Boerhaave syndrome secondary to dengue fever-related vomiting. Recognition of this rare complication and timely surgical intervention are critical to prevent fatal outcomes.

## Introduction

Boerhaave syndrome is a transmural esophageal perforation caused by severe vomiting. Alcoholism and overeating are the main risk factors. The estimated incidence is ~3.1 per million per year [1].

Morbidity and mortality are high, reaching 35%–40%, rising to 60% if treatment is delayed beyond 48 hours[1–3].

Intrathoracic perforations are particularly serious due to mediastinitis and pleural contamination. Making early diagnosis and tailored surgical strategies essential [1, 2].

Initial evaluation includes anteroposterior and lateral chest X-rays.

Water-soluble contrast esophagogram or chest/abdomen computed tomography (CT) with oral contrast remains the mainstay of diagnosis. CT is particularly useful for thoracic perforations to locate the injury, guiding the surgical approach [2, 4].

Treatment varies with patient condition, timing, and severity [4].

Non-operative management may be considered for contained perforations in stable, non-toxic patients. However, primary repair remains optimal, with success depending on early timing, patient stability, and tissue viability [2, 4].

Gastrointestinal manifestations are common in dengue fever, with severe vomiting in 54% of patients, but no cases have been linked to Boerhaave syndrome [5].

Here, we present a male patient diagnosed with dengue fever who developed Boerhaave syndrome after severe vomiting.

## Case report

A 32-year-old male patient presented with severe epigastric and left chest pain after two episodes of vomiting.

History revealed a positive dengue immunology test 1 week earlier.

He denied alcohol or heavy meal intake in the preceding 2 weeks.

On examination, he was tachypneic, tachycardic, dehydrated, and afebrile.

Chest examination revealed decreased left basal air entry with mild crepitation, abdomen showed rigidity, guarding, and mild tenderness.

Laboratory results indicated elevated inflammatory markers; arterial blood gases showed severe lactic acidosis.

**Figure 1:**
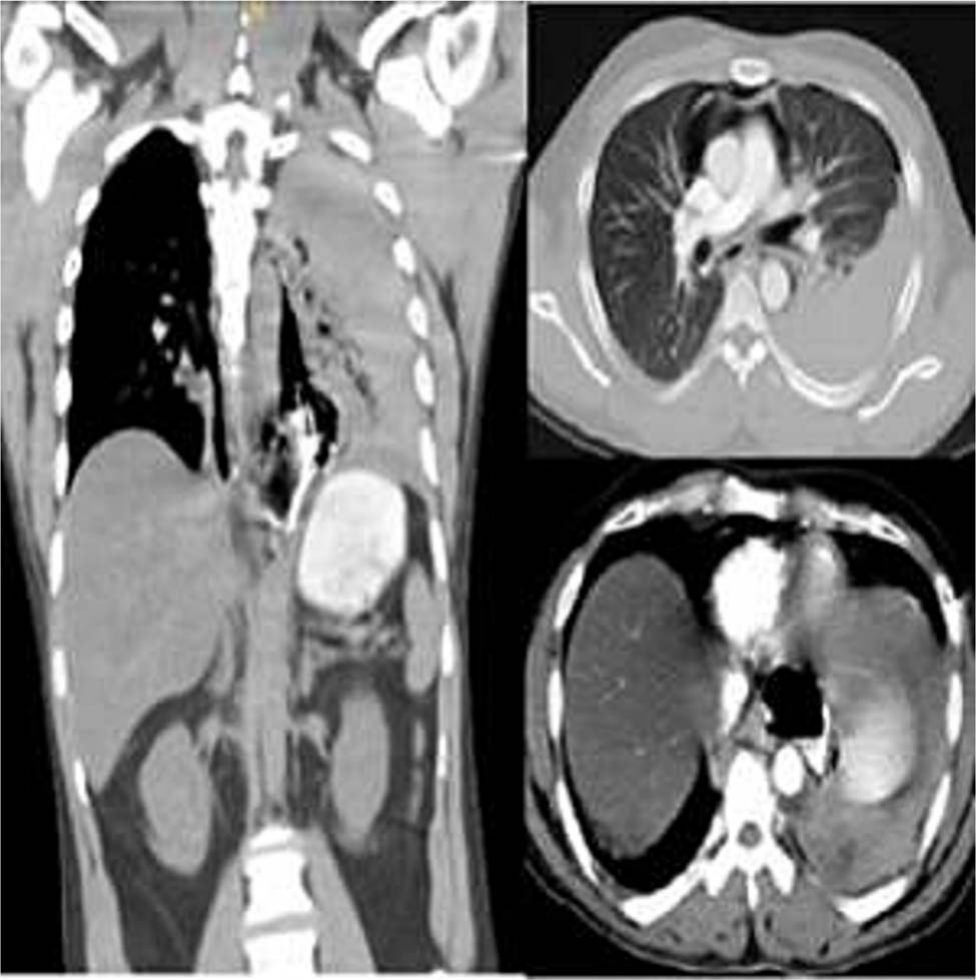
Contrast-enhanced chest CT showing distal esophageal perforation with pneumomediastinum and left hydropneumothorax.

Due to high suspicion of esophageal perforation, a contrast-enhanced chest-abdomen CT (oral and IV contrast) revealed extensive pneumomediastinum, contrast extravasation from the lower third of esophagus, and hydropneumothorax (Fig. 1). Following evaluation, Boerhaave syndrome was confirmed. The patient was stabilized in ICU, and an urgent thoracic consultation obtained.

**Figure 2:**
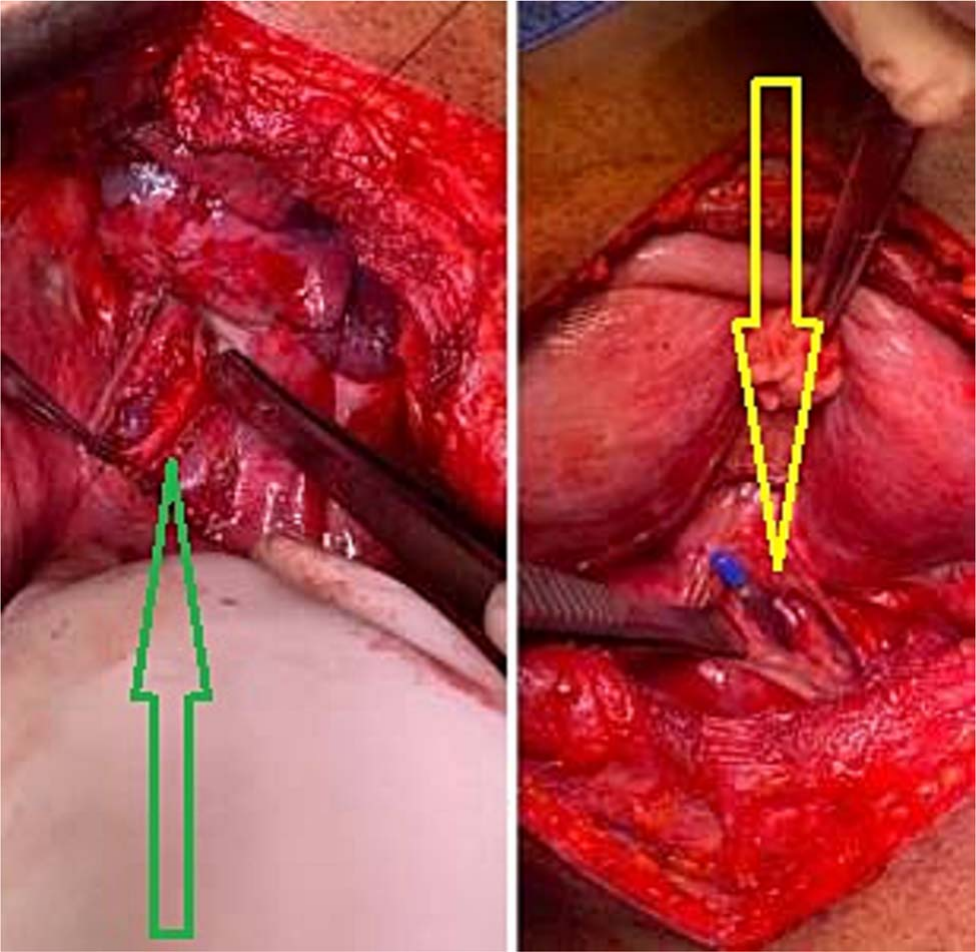
Intraoperative image demonstrating esophageal perforation (right image), and mucosal repair (left image).

A left thoracotomy revealed a 3 cm longitudinal perforation in distal esophagus, with necrotic material and turbid pleural effusion. Debridement, wide mediastinal drainage, and two-layer primary repair reinforced with an intercostal muscle flap were performed (Fig. 2).

Postoperatively, he was extubated the same day and remained hemodynamically stable, with normal lactate and urine output.

**Figure 3:**
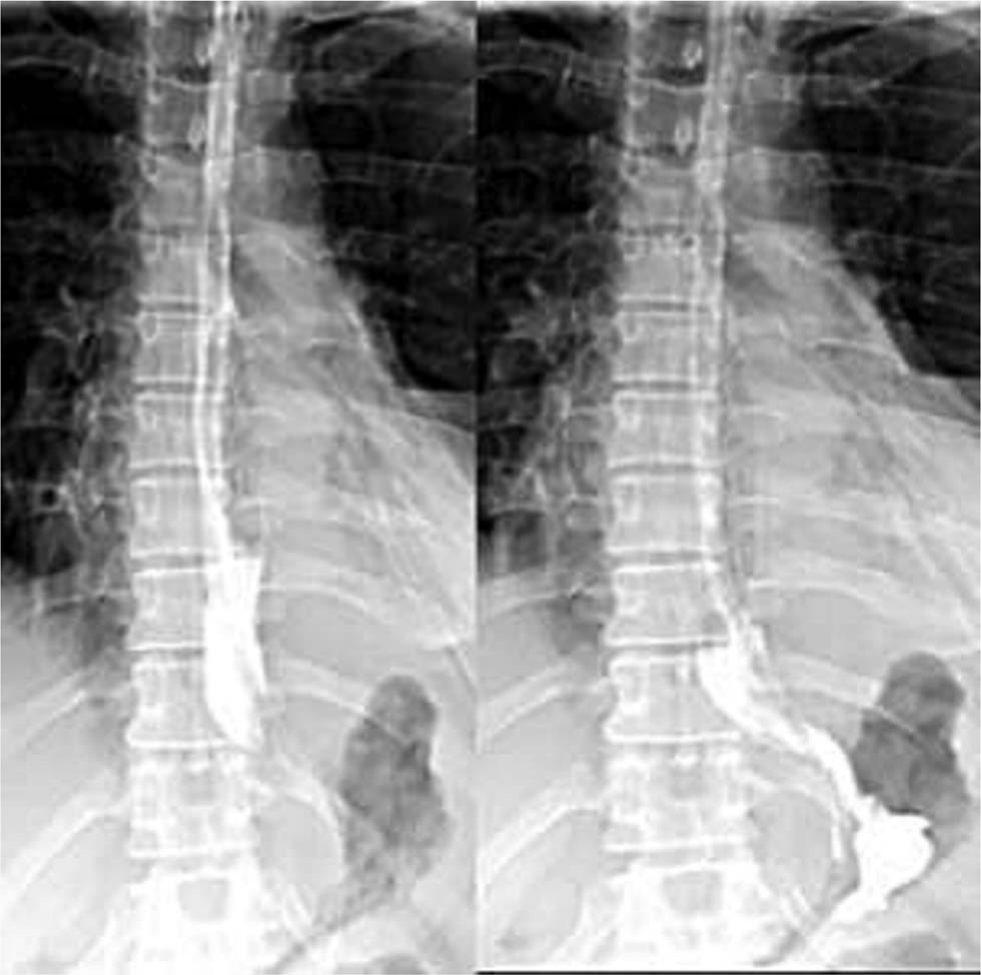
Postoperative Gastrografin esophagogram on day 5 showing free contrast flow without leakage.

Enteral feeding via nasogastric tube began on day 2 and was well tolerated. A Gastrografin esophagogram on day 5 showed free flow of contrast without leakage; (Fig. 3) the nasogastric tube was removed, and oral intake advanced. By day 7, the chest drain was removed, and the patient was discharged.

## Discussion

Boerhaave syndrome typically follows forceful vomiting.

Alcoholism and overindulgence in food are primary risk factors [3]. No published cases linked dengue fever-related vomiting to esophageal perforation.

In our patient, dengue fever preceded epigastric and chest pain after vomiting, without alcohol or heavy meal intake, representing, to our knowledge, the first documented case of Boerhaave syndrome due to dengue fever-related emesis.

The rarity of Boerhaave syndrome (0.0003% of the population) [3, 6] and atypical presentation in one-third of cases makes diagnosis challenging. Chest X-ray is sensitive but not specific; CT with oral contrast remains the gold standard for confirming and localizing perforation [3, 7].

Endoscopy may assist in inconclusive cases risks worsening the perforation and should be limited as a first-line investigation [7].

In this case, diagnosis was confirmed, so surgical management proceeded without delay.

Management depends on perforation site, timing, and patient condition. While non-operative management can be considered under strict criteria, early surgical repair offers the best outcomes [7].

Mortality is <10% if treated within 24 hours, but rises to 30%–50% thereafter [3, 7].

In this patient, young age, stable condition, and 17-hour timing favored urgent surgery, debridement, primary repair, and muscle flap reinforcement, leading to uneventful recovery.

## Conclusion

Although alcohol and heavy meals are the main risk factors for Boerhaave syndrome, rare causes such as viral infections (e.g. dengue fever) may lead to the same outcome.

Regardless of the etiology, surgical intervention remains the cornerstone of successful management and survival.

## Literature review

A comprehensive search of PubMed, Embase, Scopus, and Google Scholar up to October 2025, using the terms “Boerhaave syndrome,” “esophageal perforation,” “esophageal rupture,” “dengue fever,” and “pneumomediastinum,” revealed no prior reports of Boerhaave syndrome secondary to dengue fever–related vomiting.

This is therefore the first reported case describing this association.

## Data Availability

All data supporting the findings of this report are included within the article.
